# Immunogenicity and safety of virus-like particle of the porcine encephalomyocarditis virus in pig

**DOI:** 10.1186/1743-422X-8-170

**Published:** 2011-04-15

**Authors:** Hye-Young Jeoung, Won-Ha Lee, WooSeog Jeong, Bo-Hye Shin, Hwan-Won Choi, Hee Soo Lee, Dong-Jun An

**Affiliations:** 1 National Veterinary Research and Quarantine Service, Anyang, Gyeonggi-do, 430-824. Republic of Korea; 2 Department of Genetic Engineering, School of Life Sciences and Biotechnology, Kyungpook National University, 1370 San-Kyuk-dong, Daegu 702-701, Republic of Korea; 3 Choongang Vaccine Laboratory, Daejeon, 305-348, Republic of Korea

**Keywords:** EMCV, virus-like particles, vaccine candidate

## Abstract

**Background:**

In this study, porcine encephalomyocarditis virus (EMCV) virus-like particles (VLPs) were generated using a baculovirus expression system and were tested for immunogenicity and protective efficacy *in vivo*.

**Results:**

VLPs were successfully generated from Sf9 cells infected with recombinant baculovirus and were confirmed to be approximately 30-40 nm by transmission electron microscopy (TEM). Immunization of mice with 0.5 μg crude protein containing the VLPs resulted in significant protection from EMCV infection (90%). In swine, increased neutralizing antibody titers were observed following twice immunization with 2.0 μg crude protein containing VLPs. In addition, high levels of neutralizing antibodies (from 64 to 512 fold) were maintained during a test period following the second immunization. No severe injection site reactions were observed after immunization and all swine were healthy during the immunization period

**Conclusion:**

Recombinant EMCV VLPs could represent a new vaccine candidate to protect against EMCV infection in pig farms.

## Background

The porcine encephalomyocarditis virus (EMCV) is a member of the genus Cardiovirus of the family *Picornaviridae*, the genome is a single-stranded positive sense RNA of approximately 7.8 kb with a unique large open reading frame (ORF) [1]. Porcine EMCV infection, which is characterized by acute myocarditis and sudden death in preweaned piglets and severe reproductive failure in sows, results in severe economic losses for swine production [2-4].

An inactivated EMCV vaccine is considered as one of the effective strategies for preventing EMCV infection in domestic and wild animals [5,6]. Recently, vaccination with porcine EMCV virus-like particles (VLPs) has also been examined as a novel candidate for protection against porcine EMCV [7]. However, VLP-based vaccines against porcine EMCV produced using a baculovirus system have not yet been developed.

One of the most important technological developments to emerge from the baculovirus expression system was the observation that the expression of viral capsid proteins could lead to the assembly of VLPs that mimic the overall structure of authentic viral particles but are devoid of viral nucleic acids [8]. VLPs represent a highly effective alternative vaccine strategy. They have been shown to stimulate B-cell-mediated immune responses, and are also highly effective at stimulating CD4 proliferative responses and cytotoxic T-lymphocyte (CTL) responses [9-11]. VLPs have thus been developed as novel vaccine candidate for many kinds of viruses including bluetongue virus [12], rabbit hemorrhagic disease virus [13], severe acute respiratory syndrome (SARS) virus [14], Norwalk-like viruses [15], and parvovirus [16]. Moreover, hepatitis B virus (Recombivax HB, Merck) and human papillomavirus (Gardasil^®^, Merck) VLPs have been approved for use as vaccines.

In this study, we generated a recombinant baculovirus Bac-P12A3C, which contains the structural protein P1, the nonstructural protein 2A and the protease 3C of porcine EMCV K3 (wild strain) to induce formation of VLPs that mimic the antigenic structure of authentic porcine EMCV particles. We then evaluated the protective immune response induced by the recombinant VLPs in mice and their immunogenicity in swine.

### 2. Materials and methods

#### 2.1. Viruses, cells and antibodies

The Korean porcine EMCV K3 strain (pEMCV-K3) isolated in 1990 and the monoclonal antibody (MAb) 3F10 against the VP1 protein of pEMCV-K3 were used as described previously [7]. The Spodoptera frugiperda (Sf9) insect cells were maintained in Grace medium (Invitrogen, USA) containing 5% fetal bovine serum (Gibco, USA), lactalbumin hydrolysate (Gibco, USA), and an antibiotics-antimycotic solution (Gibco, USA) at 27°C, and infected Sf9 cells were maintained in Sf 900 II SFM (Gibco, USA) without fetal bovine serum.

#### 2.2. Construction of recombinant baculovirus transfer vectors and generation of recombinant baculovirus

Genes of the capsid protein P1, the nonstructural protein 2A and the protease 3C of pEMCV-K3 were amplified and cloned into a pFastBac™ HTB (Invitrogen, USA) as described previously [7]. The P12A3C gene was then inserted down stream of the polyhedron promoter (P_PH_). Recombinant baculovirus was generated by site-specific transposition of pFastBac/P12A3C into a baculovirus shuttle vector (bacmid) propagated in DH10Bac cells (Invitrogen, USA) by using the Bac to Bac baculovirus expression system (Invitrogen, USA) according to the manufacturer's instructions. Recombinant baculovirus (Bac-P12A3C) was plaque purified, and then the presence of the P12A and 3C genes of pEMCV-K3 was confirmed by PCR using previously described primer sets [7].

#### 2.3. Expression of recombinant proteins

Sf9 cells in 6-well culture plates were infected with recombinant baculovirus at a multiplicity of infection (MOI) of 10 for 72 h. Vero cells were infected with pEMCV-K3 grown in 6-well culture plates (as a positive control). The expressed recombinant proteins were analyzed by immunofluorescence assay (IFA) and Western blotting analysis as previously described [7].

#### 2.4. Morphology of VLPs

Sf9 cells in 25 cm^2 ^flasks were infected with recombinant baculovirus at an MOI of 10 and harvested at 4 day post-infection (dpi). The harvested cells were clarified by centrifugation, concentrated using polyethylene glycol precipitation and then, loaded onto a 20-60% (w/v) discontinuous sucrose step density gradient as described previously [7]. The peak fraction from the sucrose gradient was allowed to settle on glow-discharged carbon-coated grids for morphological examination by transmission electron microscopy (TEM, Tecnai G2) at the Korea Basic Science Institute. The grid was blotted dry, and stained with 1% uranyl acetate. The sample was visualized using a transmission electron microscope at 60,000 × magnification.

#### 2.5. Animal experiments

##### 2.5.1 Efficacy of EMCV VLPs in mice

Female BALB/c mice (aged 6-8 weeks) were used for the immunization and challenge trials. The mice were randomly divided into four groups, with fifteen mice in each group. All groups were intramuscularly (I.M) inoculated with 0.1 ml antigen two times at an interval of 2 weeks. Group 1 was inoculated with PBS as a negative control, and Group 2 was inoculated with a commercial killed vaccine (Choongang Vaccine Laboratory, Korea) as a positive control. Group 3 and Group 4 were inoculated with 0.5 μg and 0.1 μg respectively, of crude protein extract from Bac-P12A3C infected cells containing VLPs. On 14 day post-vaccination, ten mice in each group were challenged intramuscularly with 0.1 ml of the pEMCV-K3 strain containing 10^6 ^TCID_50_/ml (1000MLD_50_/ml) per mouse. The remainder in each group was analyzed for humoral immune response as described previous [17].

##### 2.5.2 Safety and Immunization in swine

Twelve pigs, weighing approximately 30-40 kg each, that were antibody - negative against EMCV were separated into six groups of two pigs each. Antigen was mixed with an equal volume of an Montandide IMS 1313™ N VG (Seppic, France), and all groups were inoculated intramuscularly with 1.0 ml antigen-adjuvant mixture. Group 1 was inoculated with PBS as a negative control, and Group 2 was inoculated with commercial vaccine as a positive control. Group 3 and Group 5 were inoculated once each with 2.0 μg and 0.2 μg of crude protein, respectively, which was extracted from Bac-P12A3C infected cells containing VLPs. Group 4 and Group 6 were inoculated twice in a 2 week interval with 2.0 μg and 0.2 μg respectively, of crude protein extracted from Bac-P12A3C infected cells containing VLPs. After each immunization, all swine were observed for 30 min and monitored for clinical signs during the immunization period at 2 day intervals. Sera were collected every week for 50 days from immunized swine to analyze seroconversion. Neutralizing EMCV antibodies were detected in collected sera using an *in vitro *neutralization assay as described previous [17].

#### 2.6. Statistics analysis

Results of neutralizing antibodies levels were presented as the means ± SEM. The significance of the variability among the experimental groups was determined by two-way ANOVA. A probability value (*P*) of < 0.05 was considered significant.

### 3. Results

#### 3.1. Expression and identification of VLPs in vitro

Sf9 cells were transfected with a bacmid contained the P12A and 3C genes, and recombinant baculoviruses (Bac-P12A3C) were identified by plaque assay and confirmed by PCR (data not shown). Cells were infected with Bac-P12A3C at an MOI of 10, and cells and supernatants were harvested and analyzed at 3 dpi, at which point most of the cells showed cytopathic effects (CPE).

**Figure 1:**
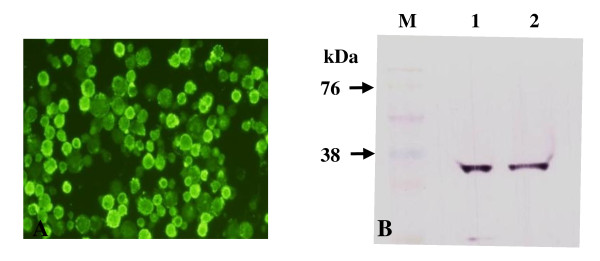
**Analysis of recombinant baculovirus expression using the monoclonal antibody 3F10, which recognizes an epitope of the VP1 protein**. (A) Expression and intracellular localization of recombinant baculovirus Bac-P12A3C as identified by immunofluorescence. (B) Western blotting analyses of recombinant baculovirus. Lane M, protein molecular weight marker; Lane 1, whole virus; Lane 2, Sf9 cells infected with Bac-P12A3C.

In the IFA, Sf9 cells infected with Bac-P12A3C reacted with the Mab 3F10 and exhibited strong cytoplasmic staining (Figure 1A). Western blotting showed that a specific protein of the predicted size of 30 kDa (VP1) was observed in both Vero cells with the pEMCV-K3 strain and in Sf9 cells infected with Bac-P12A3C (Figure 1B).

#### 3.2. Analysis of VLP morphology

**Figure 2:**
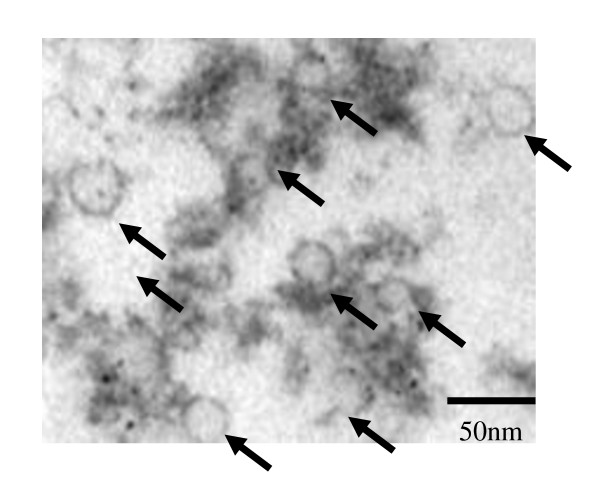
**Characterization of VLPs using transmission electron microscopy (TEM)**. TEM images of Sf9 cells infected with Bac-P12A3C. The arrows indicate aggregation of VLPs. Bar size: 50 nm

To examine whether cells infected with Bac-P12A3C can generate virus-like particles, Sf9 cells infected with Bac-P12A3C were prepared for visualization by TEM. As seen in Figure 2, hollow spherically shaped structures with a diameter of about 30 nm were identified in Bac-P12A3C-infected cells, whereas no such structures were observed in uninfected Sf 9 cells (data not shown).

#### 3.3. Seroconversion and protection of immunized mice

**Figure 3:**
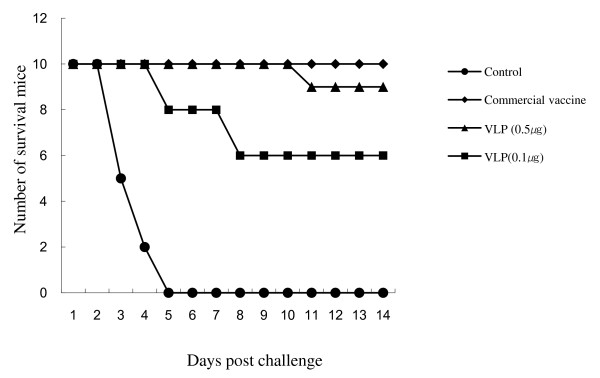
**Protection of immunized mice against a lethal EMCV challenge**. The survival curve was based on the number of mice surviving on various days post-challenge.

Serum samples were collected from each immunized mouse group on day 14 after the second immunization. The negative control group immunized with the PBS (Group 1) was negative for pEMCV-K3 neutralizing antibodies, whereas Group 2, which was immunized with a commercial vaccine, produced virus-neutralizing antibodies at high levels (from 256 to 1,024 fold). Group 3 and Group 4, which were immunized with 0.5 μg and 0.1 μg, respectively, of crude protein containing VLPs, exhibited virus-neutralizing antibodies (from 128 to 1,024 fold for Group3, and from 64 to 512 fold for Group 4). In each challenged group, all ten mice immunized with the commercial vaccine were protected perfectly, whereas mice immunized with the PBS all died due to infection within 3 to 4 days post-challenge. Nine of the ten mice immunized with 0.5 μg of crude protein containing VLPs were protected to a high level, but only 60% of the mice immunized with 0.1 μg of crude protein containing VLPs survived (Figure 3).

#### 3.4. Safety and immunity in swine

**Figure 4:**
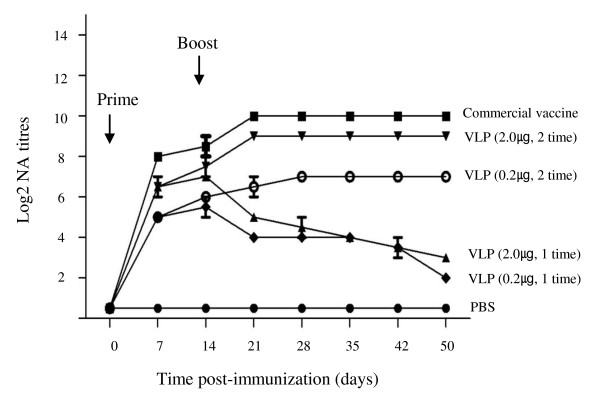
**Titers of the neutralizing antibody (NA) against the EMCV-K3 strain in the sera of immunized swine on various days post-vaccination**. The curve of NA was obtained from the average of two sera in each group. Arrows indicate the time of immunization.

As seen in Figure 4 determined by two-way ANOVA, neutralizing antibodies in the pigs of Group 3 were increased 128 fold and those of Group 5 were increased 64 fold upon inoculation with a single dose of crude protein containing VLPs. Steady production of the antibodies was maintained for two weeks, but decreased dramatically three weeks after immunization (from 4 to 8 fold). Group 4 and Group 6 showed an increased production of neutralizing antibodies after the primary and secondary inoculation with crude protein containing VLPs, and these neutralizing antibody levels were maintained at high levels for up to 5 weeks after the last inoculation. The level of neutralizing antibodies in Group 4 (from 64 to 512 fold) was similar to that of group 2. On Group 2, swine immunized with the commercial EMCV vaccine generated higher levels of virus-neutralizing antibodies with titers ranging from 256 to 1,024 fold, and these levels of neutralizing antibodies were maintained until five weeks after final inoculation. In contrast, swine in Group 1 did not produce any detectable neutralizing antibodies. No severe injection site reactions were observed after immunization and all swine were healthy during the immunization period.

### 4. Discussion

Virus-like particles are promising vaccine candidates for triggering neutralizing antibody response since they can authentically mimic the viral surface and are antigenicity similar to the parental virus. In addition, they are safely devoid of infectious genetic material [8,18]. VLPs have been produced extensively as human and veterinary vaccine candidates due to their strong immunogenicity. The baculovirus expression system has been widely used for generation of these VLPs due to the high productivity of the system and the ability to achieve rapid production scale implementation [19]. VLPs have been successfully generated from many other Picornaviridae family members, including enterovirus [20], poliovirus [21] and foot-and mouth disease virus (FMDV) [22].

In a previous study, we engineered a DNA vaccine that produced pEMCV-K3 VLPs and confirmed that the VLP antigen exhibited good antigenicity and protective immunity in mice [7]. In the present study, we used a baculovirus expression system to generate EMCV VLPs, verified that Bac-P12A3C is capable of expressing a fusion protein, and confirmed P1 protein is correctly processed by the 3C protease into the VP1 protein (approximately 30 kDa) as previously demonstrated [7,23]. These data also showed that Bac-P12A3C has the ability to self-assemble into 30 nm to 40 nm VLPs with a similar morphology to authentic virus particles of porcine EMCV showed by TEM [7,20,22,24].

Mice immunized with 0.1 μg of crude protein containing VLPs (60%), 0.5 μg crude protein containing VLPs (90%), or the commercial vaccine (100%) displayed different levels of protection in viral challenge experiments. Correspondingly, a linear relationship between the levels of neutralizing antibody and the protective efficacy of EMCV vaccines against lethal EMCV challenge has been demonstrated [7,23]. VLP-based vaccines have been shown to confer protection in animal models against many viral challenges (i.e., enterovirus [25], foot and mouth disease virus [26], influenza virus [27] parvovirus [16], human immunodeficiency [28] and rotavirus [29]. Together, these findings indicate that VLPs can be dramatically effective immunogens [8].

To assess the safety of the VLP, two doses of crude protein including VLPs were tested, with the higher dose (2.0 μg) 10-fold higher than the lower dose (0.2 μg). No immunization related clinical signs were observed in any group. After the second immunization, the levels of neutralizing antibodies were similar to those obtained with the commercial vaccine and were more effective than single-dose immunization in inducing the production and maintenance of neutralizing antibodies in swine. The twice immunization were more effective in inducing the production and maintenance of neutralizing antibodies in swine than primary immunization. Correspondingly, the conventional inactivated vaccine is also administered twice, as the second boost is needed to effectively induce neutralizing antibody [30]. The production of neutralizing antibodies has been correlated with protection against viral infection and is an important feature of an effective vaccine. In addition, some data suggest that the induction of high level of EMCV specific neutralizing antibodies may be essential for protection against EMCV infection [5,30,31]. This study demonstrates that twice immunizations with a VLP vaccine can effectively induce neutralizing antibodies. Thus, this approach may have significant application as a novel vaccine strategy to control EMCV. In future studies, we plan to investigate this VLP vaccine for efficiency, safety, and effects on litter size in pregnant swine.

Based on the results presented on this study, we conclude that porcine EMCV VLPs generated using a baculovirus expression system are safe and demonstrate good antigenicity and immunogenicity. The antigenicity from vaccination against EMCV with these VLP indicate such systems have as promising vaccines for this disease.

## Competing interests

The authors declare that they have no competing interests.

## Authors' contributions

HYJ, WHL, and DJA participated in the design and conducted the majority of the experiment the study and drafted the manuscript. WSJ and HWC performed swine experiments and analyses of data. BHS and HSL performed mice experiments and analyses of data. All authors read and approved the final manuscript.
